# Monoketonic Curcuminoid-Lidocaine Co-Deliver Using Thermosensitive Organogels: From Drug Synthesis to Epidermis Structural Studies

**DOI:** 10.3390/pharmaceutics14020293

**Published:** 2022-01-27

**Authors:** Aryane A. Vigato, Ian P. Machado, Matheus del Valle, Patricia A. da Ana, Anderson F. Sepulveda, Fabiano Yokaichiya, Margareth K. K. D. Franco, Messias C. Loiola, Giovana R. Tófoli, Cintia Maria S. Cereda, Mirela I. de Sairre, Daniele R. de Araujo

**Affiliations:** 1 Human and Natural Sciences Center, Federal University of ABC, Sao Paulo 09210-580, SP, Brazil; aryanevigato@gmail.com (A.A.V.); andersonsepulvedaster@gmail.com (A.F.S.); messiasloiola@gmail.com (M.C.L.); 2 Department of Fundamental Chemistry, Institute of Chemistry, University of São Paulo, Sao Paulo 05508-000, SP, Brazil; ianpmachado@hotmail.com; 3 Center of Engineering, Modeling and Applied Social Sciences, Federal University of ABC, Sao Bernardo 09606-045, SP, Brazil; matheusdv@gmail.com (M.d.V.); patriciadaana@gmail.com (P.A.d.A.); 4 Department Quantum Phenomena in Novel Materials, Helmholtz-Zentrum Berlin für Materialien, 14109 Berlin, Germany; fabiano.yokaichiya@gmail.com; 5 Nuclear and Energy Research Institute, Sao Paulo 01000-000, SP, Brazil; margareth_franco@yahoo.com.br; 6 São Leopoldo Mandic Research Unit, Campinas 13000-000, SP, Brazil; giovana.moniz@slmandic.edu.br (G.R.T.); cscereda@gmail.com (C.M.S.C.)

**Keywords:** organogels, poloxamer, lidocaine, curcuminoids, skin structural analysis

## Abstract

Organogels (ORGs) are remarkable matrices due to their versatile chemical composition and straightforward preparation. This study proposes the development of ORGs as dual drug-carrier systems, considering the application of synthetic monoketonic curcuminoid (m-CUR) and lidocaine (LDC) to treat topical inflammatory lesions. The monoketone curcuminoid (m-CUR) was synthesized by using an innovative method via a NbCl_5_–acid catalysis. ORGs were prepared by associating an aqueous phase composed of Pluronic F127 and LDC hydrochloride with an organic phase comprising isopropyl myristate (IPM), soy lecithin (LEC), and the synthesized m-CUR. Physicochemical characterization was performed to evaluate the influence of the organic phase on the ORGs supramolecular organization, permeation profiles, cytotoxicity, and epidermis structural characteristics. The physico-chemical properties of the ORGs were shown to be strongly dependent on the oil phase constitution. Results revealed that the incorporation of LEC and m-CUR shifted the sol-gel transition temperature, and that the addition of LDC enhanced the rheological G′/G″ ratio to higher values compared to original ORGs. Consequently, highly structured gels lead to gradual and controlled LDC permeation profiles from the ORG formulations. Porcine ear skin epidermis was treated with ORGs and evaluated by infrared spectroscopy (FTIR), where the stratum corneum lipids were shown to transition from a hexagonal to a liquid crystal phase. Quantitative optical coherence tomography (OCT) analysis revealed that LEC and m-CUR additives modify skin structuring. Data from this study pointed ORGs as promising formulations for skin-delivery.

## 1. Introduction

Organogels (ORGs) are nanostructured systems formed by three-dimensional interactions between an oil phase and an aqueous phase. Due to their versatile chemical composition, they have been considered as remarkable materials with straightforward preparation, displaying long-term stability, which allowed their wide use as matrices for pharmaceutical, cosmetic, biotech, and food technology applications [1,2,3]. The chemical compositions of the oil and aqueous phases can be diversified by using several organic solvents or oils, e.g., isopropyl myristate and palmitate, and water-soluble gelling agents, such as polymers and low-molecular-weight organogelators [4]. For instance, polymeric organogelators have being investigated in a variety of drug-delivery systems carrying small molecules, nanoparticles, and antibodies, while low molecular-weight organogelators have been used in pharmaceutical and food applications which are already approved by health authorities [3]. By modifying the chemical nature of the constituent phases, these materials can incorporate bioactive compounds with different physicochemical and pharmacological characteristics. Since they are lipophilic and easy to spread, ORGs are topically administered, where the systemic first-pass effect is avoided. In this context, the presence of an oil phase favors the ORGs interaction with the skin, since the stratum corneum (SC) lipid matrix constitutes a difficult barrier to be overcome by most formulations and drugs [5]. The challenge in topical administration of bioactive compounds is therefore to effectively promote permeation through the SC [6]. 

Recent literature proposed several formulations that aid molecules passing through the skin, such as hydrogels, nanoparticles, and emulsions [1,7,8]. Penetration enhancers are important in these materials’ composition to improve the diffusivity and solubility of molecules through the skin by reducing the stratum corneum barrier resistance. The mechanisms of penetration enhancement involve the interaction between the enhancement agents with lipids and proteins (mainly keratin) of the skin, resulting in the disorganization of stratum corneum (SC) barrier. Water, hydrocarbons, alcohols and acids can be defined as classical enhancers, while formulations containing cyclodextrin derivatives and chitosan are also widely employed [6]. For pharmaceutical applications, dual drug-delivery is a motivating topic to pursue as it is possible to combine bioactive molecules with complementary and/or synergistic action, increasing the efficiency of the topical treatment [9]. Among bioactive molecules used in skin procedures/treatments, lidocaine (LDC) is a local anesthetic widely used in clinical settings, available as commercial formulations such as gels and ointments [10]. However, such formulations have a short anesthesia duration, reducing the pharmacological effect efficiency at the site of administration [11]. Additionally, permeation-promoting organic compounds are commonly used in ORGs to disrupt the SC barrier and allowing the drugs to penetrate toward the deep layers of the skin [5,12]. 

**Figure 1:**
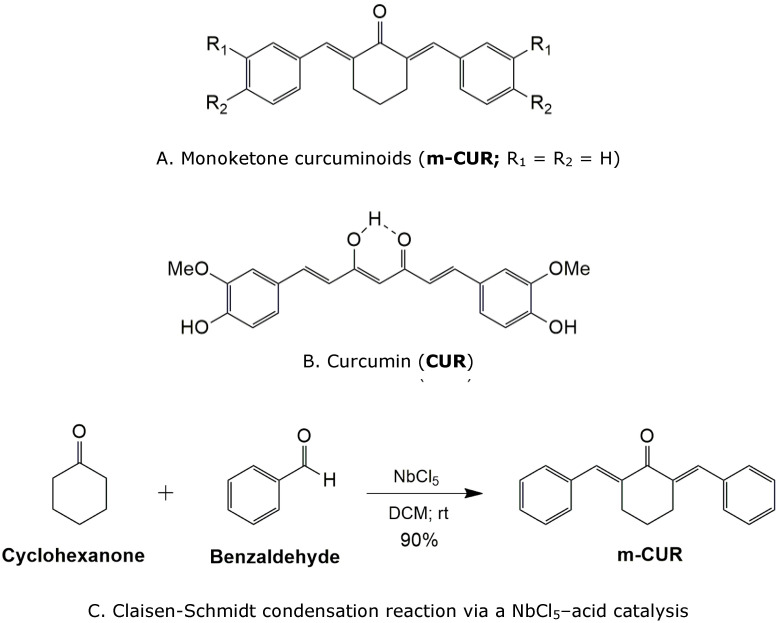
Chemical structure of (**A**) monoketone curcuminoids, (**B**) curcumin, and (**C**) a scheme of the ‘Claisen-Schmidt’ condensation reaction to synthesize the m-CUR.

Monoketone curcuminoids (m-CURs, Figure 1A) are synthetic hydrophobic compounds derived from curcumin (Figure 1B), capable of interacting with the oil phase of ORGs [13]. Due to their monoketone structure in contrast to the diketonic curcumin, m-CURs have higher chemical photostability, expanding their application potential for therapeutical formulations and extended drug-release [14,15,16]. Therefore, there is an open path within this framework to investigate ORG-based formulations for drug co-delivery, seeking synergistic effects between pharmaceutically complementary additives introduced into the different aqueous and oil phases [17]. 

In this study, ORG formulations were prepared by incorporating an oil phase, composed of isopropyl myristate (IPM) and soy lecithin (LEC), into an aqueous phase of thermosensitive poloxamer PL407 [18,19,20]. The monoketone curcuminoid (m-CUR) was synthesized by a Claisen-Schmidt condensation reaction via a NbCl_5_–acid catalysis, and it was added as the bioactive molecule of the oil phase (Figure 1C) [15]. Aiming at co-delivery applications, lidocaine hydrochloride (LDC) was incorporated into the ORGs aqueous phase. A thorough physico-chemical characterization was carried to assess the curcuminoid influence on ORG structural organization, as well as on the local anesthetic permeation and cytotoxicity. In addition, the interaction of ORGs with SC lipids was evaluated by infrared spectroscopy (FTIR) and optical coherence tomography (OCT) to investigate the epidermis structural and morphological changes after treatment with ORGs [21,22]. 

In this context, our study targets at the development of m-CUR/LDC-activated ORGs and at investigating the influence of their components on the drug permeation profiles, epidermis structure, and cytotoxicity, aiming at the treatment of skin inflammatory diseases [23,24]. 

## 2. Materials and Methods

### 2.1. Chemicals and Reagents

Reagents and solvents used for monoketone curcuminoid synthesis were obtained from commercial sources. NbCl_5_ was supplied by Companhia Brasileira de Metalurgia e Mineração (CBMM, Araxá, Minas Gerais, Brasil). Thin layer chromatography (TLC) was performed on silica gel matrix (Sigma-Aldrich Chem. Co., San Luis, MO, USA) by using pre-coated plates containing fluorescein as indicator (λ = 254 nm). ^1^H and ^13^C NMR spectra were recorded on Varian equipment (500 MHz, Palo Alto, CA, USA) using deuterated chloroform solvent (CDCl_3_, Sigma-Aldrich Chem. Co., San Luis, MO, USA). High-resolution mass spectra (HRMS) were measured on a micrOTOF-Q II (Bruker Daltonics, Billerica, MA, USA).

Pluronic^®^ F-127 (PL407) and lidocaine hydrochloride (LDC) were purchased from Sigma-Aldrich (San Luis, MO, USA). Isopropyl myristate (IPM) and soy lecithin (LEC) were obtained from Dinâmica (São Paulo, Brazil). All other chemicals and solvents were analytical grade.

### 2.2. Synthesis of 2,6-Dibenzylidenecyclohexanone (m-CUR)

For the m-CUR synthesis, benzaldehyde (3 eq.), cyclohexanone (1 eq.), and niobium pentachloride (NbCl_5_, 1.5 eq.) in dichloromethane were added to a round bottom flask fitted with a drying tube. The reaction mixture was stirred at room temperature. The reaction was monitored by TLC until practically total consumption of cyclohexanone was verified (5 min). Then, the mixture was dissolved in dichloromethane and filtered through silica gel to remove the catalyst. The filtrate was concentrated on a rotary evaporator and the pure product as a yellow solid was obtained after recrystallization from methanol and drying in a desiccator (90% yield). ^1^H NMR (500 MHz, CDCl_3_) δ: 7.83 (2H, s); 7.49 (4H, m); 7.42 (4H, m); 7.36 (2H, m); 2.95 (4H, t, *J* = 6.2 Hz); 1.80 (2H, quint., *J* = 6.2 Hz). ^13^C NMR (CDCl_3_, 125 MHz) δ: 190.27 (C); 136.86 (CH); 136.16 (C); 135.95 (C); 130.30 (CH); 128.52 (CH); 128.32 (CH); 28.39 (CH_2_); 22.96 (CH_2_). HRMS *m*/*z* [M + H]^+^ calcd: 275.1430; found: 275.1450.

### 2.3. PL-Based Organogels Preparation

Firstly, the oil phase (OP) containing 2 mL isopropyl myristate (IPM) and the additives, LEC (2% *w*/*v*) and m-CUR (1 mg/mL), was prepared under magnetic stirring in a water bath (~60 °C) until homogenization. The aqueous phase (AP), consisting of PL407 (30% *w*/*v*) and sodium benzoate/methylparaben/propylparaben (0.25, 0.1, and 0.05% *w*/*v*) as preservatives, was prepared in an ice bath under magnetic stirring (300 rpm). Then, LDC (25 mg/mL) was added to AP. After solubilization, the AP was kept at 8 °C until use. To finally prepare the ORG formulations, OP was added to AP (1:4 *v*/*v*) and mixed with a glass rod until obtaining a homogeneous system. The final concentrations of m-CUR and LDC were 0.02% and 2% (*w*/*v*), respectively, for all formulations. The final ORGs formulations were (Table 1): ORG, ORG-LDC, ORG-LEC, ORG-LDC/LEC, ORG-LEC/m-CUR, and ORG-LDC/LEC/m-CUR, where the ORG formulation (control) consists of PL407 30% *v*/*v* (aqueous phase) and IPM (oil phase) [11,25].

### 2.4. Organogels Physico-Chemical Characterization

#### 2.4.1. Organoleptic, pH and Morphological Characterization

The ORGs were assessed by visual inspection of color, odor, phase separation, and aggregate formation. The pH of every ORG was recorded with a pH meter after electrode equilibrium. Morphological analysis of the ORGs was performed by using an FEI Quanta 250 scanning electron microscope (SEM). The ORGs were spread on a microscope slide forming a thin film and dried in a desiccator for 24 h. The resultant dry powder was deposited on carbon tape, which was previously attached to an aluminum stub. SEM images were acquired under electron acceleration voltage of 5 kV and image magnification of 196×.

#### 2.4.2. Differential Scanning Calorimetry (DSC)

Calorimetric analyses were performed using a Netzsch DSC Polyma calorimeter (Netzsch, Selb, Germany). The ORG formulations were weighed in hermetic aluminum pans (20 mg) and measured under three thermal cycles (heating–cooling–heating) from 0 to 50 °C at 5 °C/min ratio. All measurements were executed in triplicate. Thermogram data were represented by the heat flux (J/g) versus temperature (°C). An empty aluminum crucible was used as the reference.

#### 2.4.3. Rheology

Rheological analyses were performed using a cone-plate type geometry in an oscillatory rheometer (Kinexus Lab., Malvern Instruments, Malvern, UK). The ORGs were analyzed in the 0–50 °C temperature range at 1 Hz frequency to determine the sol-gel transition temperature (T_sol-gel_). Oscillatory analyses were performed under variable frequency interval (0.1–10 Hz) at 32.5 °C, for correlation tests related to the elastic module (*G′*), viscous module (G″) and apparent viscosity (η*). The relation between *G′* module and oscillation frequency (ϖ) was used to calculate the ORG′s strength using Equation (1):

(1) $$G^{\prime }=S\;\cdot \;\varpi^{n}$$

where *S* is the formulation strength (Pa·s) and *n* is the viscoelastic exponent.

#### 2.4.4. Small Angle Neutron Scattering (SANS)

SANS measurements were performed using the V16 instrument at Helmholtz-Zentrum Berlin (HZB). The scattering data were recorded at two different distances from the detector: 2 m (wavelength 1.8–3.8 Å) and 11 m (wavelength 1.6–9.2 Å), covering the 0.007–0.5 Å^−1^ q interval. Organizational samples were prepared in D_2_O, arranged in quartz cubes (1 mm optical path), and measured at 25 and 40 °C. The MANTID data reduction package, adapted for V16, was used to process the generated data.

### 2.5. In Vitro Permeation Experiments

In vitro permeation tests were carried out by a vertical diffusion cells system with a permeation area of 1.72 cm^2^ (Microette Plus; Hanson Research, Chatsworth, CA, USA). The donor and recipient compartments were separated by an artificial membrane that mimics the organization of the human skin (Strat-M^®^) [26]. The receptor compartment was filled with 7 mL of water/ethanol 70:30 *v*/*v* solution and kept under constant magnetic stirring (350 rpm) at 32.5 ± 0.5 °C. At regular intervals (0.5 to 48 h), aliquots from the receptor compartment were collected and then analyzed by HPLC. LDC cumulative amounts were expressed as μg·cm^−2^. Drug flux values were obtained from the slope of the curves over the 24-h period. Data were analyzed by the following Equation (2):

(2) J=P·Cd

where *J* (μg·cm^−2^·h^−1^) is the drug flux through of the membrane, *P* (cm·h^−1^) is the permeability coefficient, and *Cd* (μg·cm^−2^) is the drug concentration in the donor compartment. The lag time was calculated by extrapolating the time axis in the graph [27].

### 2.6. LDC and m-CUR Chromatographic Conditions

LDC concentrations were quantified by a HPLC system (Ultimate 3000, Chromeleon 7.2 software, Thermo Fisher Scientific, Waltham, MA, USA) coupled to DAD detector, and to a C18 column (150 × 4.6 mm, 5 μm; Phenomenex, Torrance, CA, USA) at 30 °C. LDC samples were analyzed with a 0.6 mL/min flow, using a 20:80 mixture of acetonitrile and acetic acid solution 0.05% *v*/*v* as the mobile phase. Drug retention time was 3.5 min. The limits of detection (LD) and quantification (LQ) were determined from a previous standard curve of LDC at 20, 25, 50, 60, 80, 100, 200 and 250 μg/mL. LD and LQ values found were 3.31 and 9.73 μg/mL, respectively, and LDC concentration was derived from the equation y=0.0244x+0.0011 (R^2^ = 0.998). For the m-CUR molecule, the employed flow was 0.8 mL/min, using a mobile phase of 90:10 mixture of acetonitrile and acetic acid solution 0.05% *v*/*v*. Molecule retention time was 4.7 min. LD and LQ values found were 0.34 and 1.03, respectively, and the m-CUR concentration was derived from the equation y=0.7483x+0.0054 (R^2^ = 0.995) in concentrations between 5 and 100 μg/mL. All methods followed the recommendations from the International Conference on Harmonization, and the results represent three experiments performed in triplicate.

### 2.7. In Vitro Cell Viability Assays

HaCaT epidermal keratinocytes (Thermo Fisher Sci., Waltham, MA, USA) were seeded in 96-wells plates (2.104 cells/well) for 48 h under humidified atmosphere (37 °C and 5% CO_2_), using DMEM (Gibco Laboratories, Grand Island, NY, USA) supplemented with 10% (*v*/*v*) fetal bovine serum (pH 7.2–7.4) and 100 μg/mL of penicillin/streptomycin. Predetermined amounts of ORGs were homogenized in DMEM by using a vortex for 5 min., in a concentration interval from 0.005 to 0.7 mg/mL (corresponding to 0.001 to 0.14 and from 1 to 140 µg/mL of m-CUR and LDC, respectively), and were used for treating cells during 24 h. For cell viability determination, Methylthiazolyldiphenyl-tetrazolium bromide (MTT) solution (100 µL, at 5 mg/mL in solution in phosphate buffered saline) was added to wells and incubated for 4 h. After this period, MTT solution was removed, and DMSO was added (50 μL) for 10 min. Absorbance measurements were acquired at 570 nm. Non-toxic control wells were treated with DMEM at the same conditions used for ORGs formulations.

### 2.8. Epidermis Structural Analysis

#### 2.8.1. Porcine Ear Skin Preparation

Porcine ear samples were obtained from a local slaughterhouse and the experimental protocol was approved by the UFABC Institutional Committee for the Care and Use of Animals (#8719010318). Blood vessels and subcutaneous tissue were extracted, obtaining a 2 mm-thick structure which was then dermatomed (0.45 mm, Nouvag, Rorschach, Switzerland) to completely remove the subcutaneous tissue. The dermatomed skin was immersed in a water bath at 60 °C for 3 min, and then the isolated epidermis was detached using a spatula. The epidermis samples were stored at −20 °C for a maximum of three months. For structural analysis, epidermis was treated with ORG formulations on a delineated area of 1.72 cm^2^ (~0.6 g of ORG) and kept in contact for 24 h at room temperature [27]. Finally, formulations were removed with the aid of a soft spatula, and the treated epidermis were analyzed by FTIR and OCT. Formulations containing the drug of interest (LDC) were excluded from this study in order to investigate the influence of the ORG matrix on the structure of the stratum corneum, without LDC interference.

#### 2.8.2. Fourier Transform Infrared Spectroscopy (FTIR)

Fourier transform infrared spectra were obtained for ORG individual components, ORG formulations, and epidermis samples (1 cm^2^) before and after formulations treatment. FTIR spectra were recorded in the 4000–650 cm^−1^ range with 1 cm^−1^ resolution using a PerkinElmer Spectrum Two 160,000A on ATR mode [21,28,29].

#### 2.8.3. Optical Coherence Tomography (OCT)

In vitro cross-sectional tomographic images of ORG-treated epidermis were performed using an Optical Coherence Tomography device (Fourier domain) Callisto110C1 (ThorLabs Inc., Newton, NJ, USA). Epidermises surfaces (1 cm^2^) were treated with different ORG formulations (10 mg) for 4 and 24 h, keeping the samples in closed flasks at room temperature. Non-treated epidermises samples were used as a control for all image scans.

The OCT device operates at a central wavelength of 930 nm, with axial resolution of 7 µm, transverse resolution of 8 µm, and maximum penetration depth of 1.71 mm in air. The system was programmed to automatically save the images in transmission mode, which presented numerical matrices of 1497 columns (width) × 449 lines (depth). In tomographic images, each column corresponds to an A-scan, and the pixel resolution was 3.23 µm × 3.23 µm. 

Epidermis samples were individually positioned perpendicular to the light beam on an X-Y-Z micrometric control platform. Each sample was scanned in the central region (B-scan), resulting in a sectional image (tomogram) composed of several A-scans (columns of pixel intensity) aligned side by side. B-scans were registered in triplicate to reduce speckle noise, being composed of numerical matrices of intensities in dB with the files being saved in *.oct and *.png format.

Acquired data were analyzed using an algorithm developed in MATLAB (MathWorks Inc., Natick, MA, USA) adapted for skin samples. The background was subtracted from each B-scan, and then a low pass filter was applied to minimize speckle noise. Ten A-scans (regions of interest, ROIs) manually selected and equidistant from each other were analyzed from each B-scan, which started at the pixel with the highest intensity located on the sample surface. ROIs were carefully positioned in areas not obstructed by large signs of hyporeflection, such as the presence of hair.

Image linearization and consequent signal normalization was performed using the pixel with the highest surface intensity as a reference. For this, all previous pixels were erased reading from top to bottom of the image, i.e., from the surface to the depths of the sample, which contributed to reduce the Fresnel reflection. Therefore, each A-scan was normalized by the maximum intensity value and the depth was adjusted considering a further 77 subsequent points (248.38 µm) for the dermatomed skin samples.

Two parameters were calculated from each A-scan: the optical attenuation coefficient (*µ*) and the integrated reflectivity (ΔR). To calculate the *µ*, a modified equation based on the Beer–Lambert Law was used Equation (3), where the *I*(*z*) is the intensity as a function of depth (*z*) [30].

(3) $$I\left(z\right)=I_{0}e^{\left(-2\mu z\right)}$$

The integrated reflectivity (ΔR) was calculated from the area under the A-scan profile, and the depth was adjusted by the number of points as described in the μ calculation. Finally, the area under each A-scan curve was integrated and the ΔR was given by the average of the obtained areas [31].

### 2.9. Statistical Analysis

Data were expressed as the mean ± standard deviation and analyzed by one-way analysis of variance (one-way ANOVA) using a Tukey–Kramer test. All data were analyzed by Graph Pad Prism (Graph Pad Software Inc., San Diego, CA, USA), and statistical differences were defined as ** *p* < 0.05 or * *p* < 0.1.

## 3. Results

### 3.1. Synthesis and Structural Characterization of the Monoketone Curcuminoid

Curcumin exhibits a wide variety of biological activities and is considered a natural anti-inflammatory [16,24]. However, it has the disadvantage of low stability and bioavailability due to the β-diketone moiety. Some synthetic monoketone curcumin analogues are more stable than their parent molecules, being potential anti-inflammatory pharmacophores [32]. Monoketone curcuminoids can be obtained from the Claisen–Schmidt condensation reaction between aromatic aldehydes and ketones. In general, such reactions are carried out in a strongly basic medium; however, the presence of hydroxyl groups in the aromatic ring (for example) can promote parallel reactions [33]. Here, the monoketone curcuminoid m-CUR was synthesized by acid-catalyzed Claisen–Schmidt condensation using cyclohexanone and benzaldehyde (Figure 1C). The synthesis of m-CUR was performed using niobium pentachloride (NbCl_5_) as a Lewis acid catalyst that spontaneously releases hydrochloric acid into the reaction medium. According to the mechanism, NbCl_5_ favors the reaction by increasing the reactivity of the aldehyde due to its action as a Lewis acid, in addition to favoring the enol form of the keto-enolic balance of cyclohexanone. 

Niobium compounds are most commonly used as efficient Lewis acids, where the niobium pentachloride (NbCl_5_) is rapidly hydrolyzed on contact with moisture to become HCl and Nb_2_O_5_·*n*H_2_O [34]. Thus, the decomposition of NbCl_5_ assisted the formation of the desired m-CUR product. A variety of applications of NbCl_5_ in organic synthesis have been reported, for example, in Diels–Alder reactions, multicomponent reactions (MCR), and one-pot reactions, among others [35,36]. In this context, the use of NbCl_5_ consists of a new alternative to Claisen–Schmidt condensation. All procedures in the synthesis of m-CUR were shown to be efficient, easy to perform and safe.

The m-CUR product was obtained in 90% yield and the structure was confirmed by nuclear magnetic resonance spectroscopy (NMR). ^1^H-NMR spectrum (Figure S1) confirmed the cyclohexanone ring at δ 1.80 ppm (2H, quintet) and δ 2.95 ppm (4H, triplet). The β hydrogen of the α-β unsaturated ketone was seen as a singlet at δ 7.83 ppm and aromatic hydrogen signals were in the δ 7.30–7.50 ppm region. The ^13^C-NMR spectrum (Figure S2) also confirmed the structure; the carbon signal referring to the carbonyl group was identified at δ 190 ppm with a characteristic chemical shift to α-β unsaturated ketone.

### 3.2. Organoleptic, pH, and Morphological Characterization

**Figure 2:**
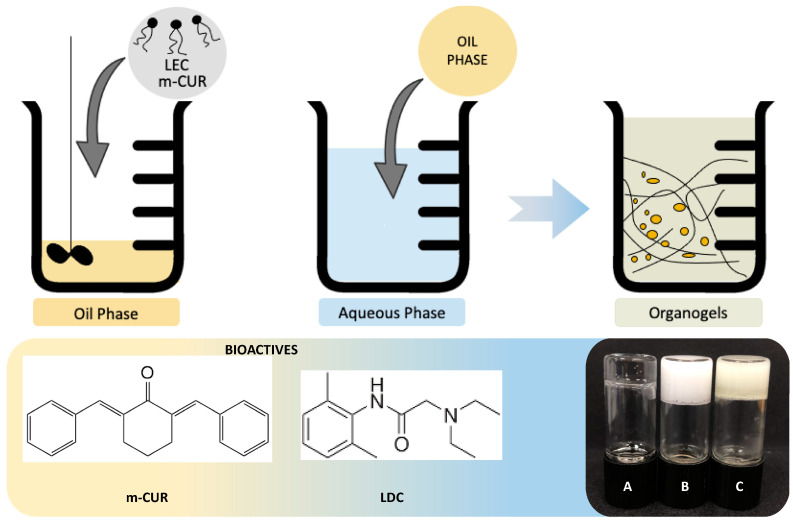
PL-based organogels preparation scheme. Inserted pictures below show (**A**) PL407 hydrogel, (**B**) PL407 + IPM organogel (ORG), and (**C**) m-CUR-loaded PL407 + IPM ORG.

All synthesized ORGs were essentially odorless and highly viscous (Figure 2). PL407 hydrogels were transparent (Figure 2A), while the ORGs exhibited white color due to the organic phase incorporation (Figure 2B), which turn yellowish after the m-CUR addition (Figure 2C). No phase separation was observed during the six-month period in which samples were stored at room temperature, protected from light. Measured pH values ranged from 5.2 to 6.3 for ORGs containing LDC and LEC, respectively. Such pH values were similar to other PL-based ORG systems containing lanolin, which were described to be adequate for skin application [11,37].

**Figure 3:**
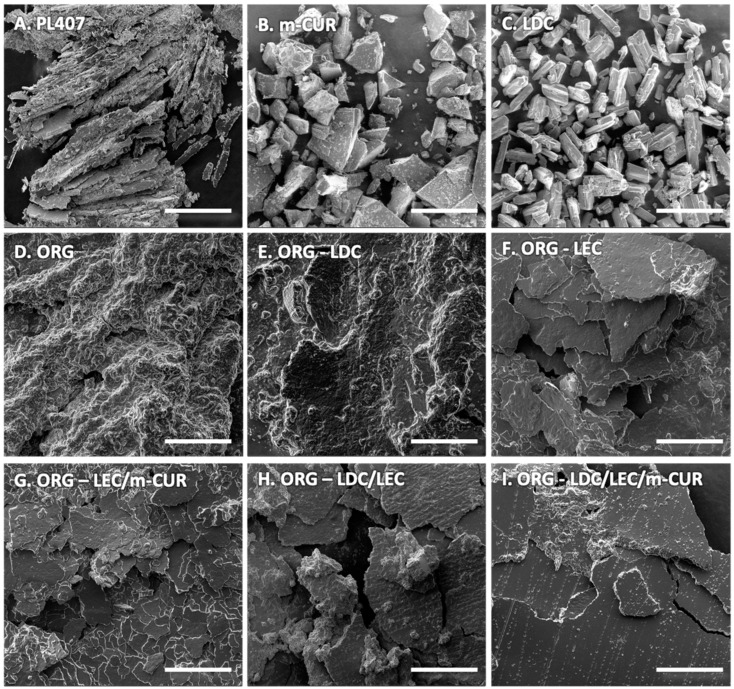
Representative scanning electron micrographs of the (**A**) PL407 hydrogel, (**B**) m-CUR, (**C**) LDC, (**D**) ORG, (**E**) ORG-LDC, (**F**) ORG-LEC, (**G**) ORG-LEC/m-CUR, (**H**) ORG-LEDC/LEC, and (**I**) ORG-LDC/LEC/m-CUR. Scale bar = 500 μm.

The morphology of all synthesized ORGs was evaluated by scanning electron microscopy (Figure 3). SEM images confirmed the typical layered arrangement for the dried PL407 hydrogel (Figure 3A) as described by Akkari et al. [38]. The control ORG and the ORG-LDC (Figure 3D,E) displayed a shapeless character, exhibiting wrinkled surfaces, as reported in the literature [11,39]. On the other hand, the other ORG samples (Figure 3F–I) showed intermediate morphology between the control ORG and the PL407 hydrogel, i.e., the layered structure was more preserved. All ORG formulations were shown to be homogeneous, without the presence of drug crystals (Figure 3B,C), indicating the systems homogeneity and incorporation of additives into the ORG matrix [40].

### 3.3. DSC Analysis

All ORGs were analyzed by DSC to determine their initial (T_onset_), peak (T_peak_), and final (T_endset_) phase transition temperatures, in addition to the micellization enthalpy (ΔH_m_) values. The analyses did not reveal changes in the values of phase transition temperature (T_peak_) in response to the incorporation of additives (Table 2). T_onset_ and T_endset_ values were also similar among the ORGs. The ΔH_m_ values given by the integration of the endothermic peak were also quite similar among the samples, but relatively lower for the ORG samples containing m-CUR and LEC. This result indicates that the PL407 micellization process was facilitated by the formation of new intermolecular interactions with the presence of LEC and m-CUR into the organic phase. In this way, the presence of additives increased the stability of the ORGs [41].

Aqueous solutions containing 20–30% of PL407 turn into gel close to body temperature due to the dehydration of the hydrophobic units in such temperature. This dehydration process enhances the interactions between the hydrophobic units, promoting the formation of PL micelles [42]. The micellization temperature (T_peak_) in PL-based ORGs is affected by the chemical characteristics of the oil phase, tending to be lower than the T_peak_ values observed for the corresponding hydrogels [39,43]. Furthermore, previous studies showed that the addition of the hydrophobic molecules in ORGs increases the micellization enthalpy [11]. The opposite behavior was observed in this work, i.e., adding LEC and m-CUR into the PL407 + IPM ORGs leads to a decrease of the micellization enthalpy values: ORG 8.3, ORG-LEC 8.0, and ORG-LEC/m-CUR 7.4 J·g^−1^ (Table 2). Nevertheless, this effect was less pronounced for PL407 + IPM ORGs. This fact can be due to the fact that LEC and m-CUR molecules, probably, act as stabilizers in the intermicellar space, decreasing the necessary energy to form PL-based micelles via intermolecular interactions in the hydrophobic–hydrophilic interface present in the ORGs.

### 3.4. Rheology Analysis

The investigation of rheological properties of drug-carrier ORG systems is essential once the shear rate and the viscosity deeply affect the drug-release rate from the formulation complex structure [44]. A thorough rheological characterization is also able to investigate the interactions between different components in a formulation, analyzing parameters such as phase-transition temperature and shear stress. Exploring these parameters leads to a deeper understanding of the formulations structural and physicochemical properties, which is crucial to further guarantee the quality of the ORGs regarding spreadability and long-term stability [45]. In addition, modulating the drug release towards slower rates is interesting once it reduces the reapplications procedures needed and decrease direct contact with the wound, improving patient compliance [46,47].

**Figure 4:**
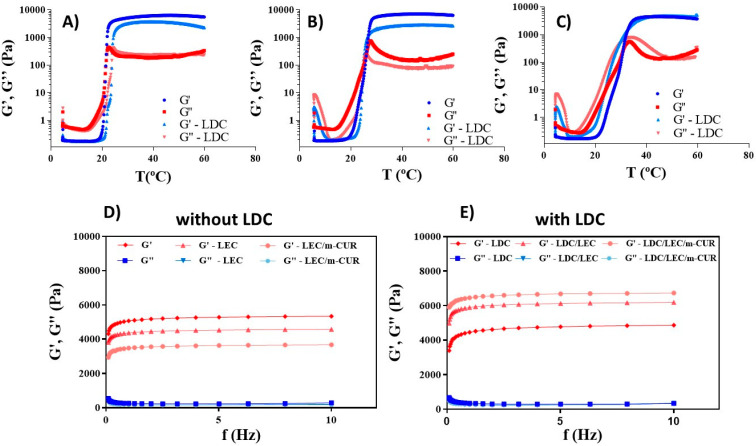
Representative sol–gel transition temperature rheograms: (**A**) ORG and ORG-LDC, (**B**) ORG-LEC and ORG-LDC/LEC, and (**C**) ORG-LEC/m-CUR and ORG-LDC/LEC/m-CUR; rheological frequency sweep analysis for the ORGs formulations (**D**) without LDC and (**E**) with LDC.

In this sense, the following rheological parameters were recorded for all prepared ORG samples (Table 3 and Figure 4): sol–gel transition temperature (T_sol-gel_), elastic modulus (G′), viscous modulus (G″), and the apparent viscosity (η*). The sol–gel transition temperature (Figure 4A–C) gives an estimative of the structural influence of the oil phase and the different additives in the PL407 gel arrangement. T_sol-gel_ was defined as the temperature point where the G′ and G″ module values were equal [48]. It was observed that the T_sol-gel_ is lower than the skin temperature (32.5 °C) for all prepared ORGs, which indicates the high stability of these biomaterials regarding phase separation for topical application. Moreover, the ORGs containing additives exhibited higher T_sol-gel_ compared to the control ORG. This increase in temperature was found more pronounced for the formulations containing m-CUR. Probably, the oil phase of IPM and its additives m-CUR and LEC increase the intermolecular distances between poloxamer micelles, avoiding their interaction. Thus, a higher energy is required to start the gelation process. In this way, m-CUR and LEC additives decrease the enthalpy for the PL407 micelle formation, as observed from the DSC results (Table 2), but they increase the energy involved in building the three-dimensional gel network originated from the interaction between these micelles (Table 3). Literature reports T_sol-gel_ values around 9.5 °C for PL407 30% hydrogels containing 2% LDC hydrochloride, where the addition of the drug did not change the T_sol-gel_ temperature [44]. From our results, it can be concluded that the oil phase is therefore responsible for the increase on T_sol-gel_ of PL407-based ORGs.

Frequency scan analysis revealed a G′ modulus superior to the G″ modulus for all ORGs, indicating a predominant elastic behavior. According to the literature, for gels with G′ > G″ across the whole experimental frequency range, the moduli should be essentially frequency independent, precisely as observed in Figure 4D,E [49]. The m-CUR and LEC oil phase additives contributed to enhance the calculated G′/G″ ratio, meaning an increase in the structural organization degree of the ORGs when compared to the control ORG. Regarding the presence of LDC in ORGs, the G′/G″ ratio was lower when compared to the respective formulation without LDC, with the ORG-LDC/LEC/m-CUR formulation being the only exception to this behavior.

Lidocaine hydrochloride (LDC) is a hydrophilic drug; therefore, it interacts mainly with the outer hydrophilic region of the PL407 micelles. On the other hand, m-CUR is a hydrophobic molecule, and LEC is an amphiphilic phospholipid with relatively long carbon chains. When combined, the possibilities for new intermolecular interactions (hydrophobic, dipole-dipole and hydrogen bonds) increase between the PL network and these molecules, contributing to a higher degree of gel structuring [39]. This reflects the increase in viscosity values and G′ modulus, particularly for the ORG-LDC/LEC/m-CUR formulation, where all additives are present.

### 3.5. SANS Analysis

Small-angle elastic neutron scattering (SANS) is a powerful tool to investigate the structure of biomaterials at intermediate dimensions between the bulk and the nano scales. The ability of poloxamer and lipids to form organized structures of different symmetries (e.g., cubic, hexagonal) in aqueous dispersions, has been known for decades [50]. In order to study their organizational structure, the PL407 + IPM-based ORGs were characterized by using the SANS technique, measuring the samples at 40 °C, i.e., after the sol–gel transition for all ORGs (Table 4). All SANS patterns are displayed of Figures S3 and S4 (Supplementary material), for 25 °C and 40 °C, respectively. 

From the SANS results, it was possible to assign two different cubic phases coexisting in all ORG samples, the $Pm\bar{3}n$ (space group number 223) and the $Fd\bar{3}m$ (space group #227), both corresponding to face-centered cubic (FCC) arrangements. No difference in the 1/q values was observed for all samples when comparing the two different temperatures, meaning that the gels were already structurally organized at 25 °C. In addition, it is worth mentioning that the lattice parameters remained practically unchanged for the different formulations, corroborating the discussion on the rheology results, i.e., the oil phase is the main responsible for the structural changes in the PL407 + IPM gel organization, while the additives have little to no influence [51]. This is further evidenced by the SANS results for the ORG-LEC formulation, the single case of different lattice parameters for the $Pm\bar{3}n$ and $Fd\bar{3}m$ structures.

One interesting result is the presence of a third cubic phase for the ORGs containing two or more additives. The $Ia\bar{3}d$ phase (space group #230) was detected for the ORG-LDC/LEC, ORG-LEC/m-CUR, and ORG-LDC/LEC/m-CUR samples, exhibiting the sample lattice parameters values for both measured temperatures. There is still some debate on the three-dimensional structure of highly concentrated PL407-based hydrogels. While Wu et al. suggested a face-centered cubic (fcc) structure, Li et al. discussed a cubic packing of spherical micelles [52,53]. In the latter, solvent molecules would play an important role on regulating the intermicellar space structural organization. Such results are not conflicting with the SANS results of this work, since the physico-chemical properties of the ORGs were shown to be strongly dependent on the oil phase constitution. For instance, our results demonstrated that LEC is an important compound to the structural organization of PL407 + IPM micellar gels. Further small angle X-ray scattering experiments will be carried out to investigate the contribution of each cubic phase to the overall structure of the ORGs.

### 3.6. In Vitro Permeation and Cell Viability Assays

**Figure 5:**
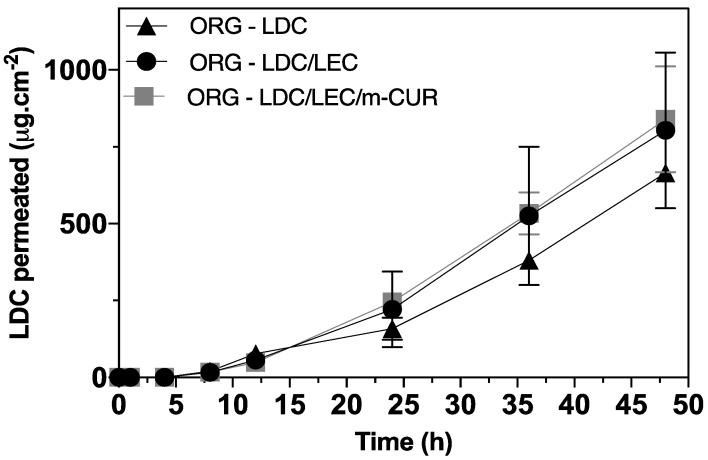
Permeation profiles of lidocaine hydrochloride (LDC) from ORG-LDC, ORG-LDC/LEC and ORG-LDC/LEC/m-CUR across the Strat-M^®^ membrane. Data expressed as mean ± standard deviation, *n* = 6/experiment.

In vitro permeation studies were performed across the Strat-M (Merck Millipore, Darmstadt, Germany), a synthetic membrane with a lipid-impregnated porous structure, which provides similar properties compared to those of animal models and human skin [26,54]. For all ORGs, permeation profiles through the Strat-M revealed LDC concentrations increasing cumulatively during the 48 h-experiment (Figure 5). The permeation flux, latency time, and permeation coefficient were then calculated (Table 5). In general, the LDC permeation profile was shown to be similar for all formulations, with no significant statistical difference between different formulation compositions. The obtained LDC flux values were comparable to those previously reported by our group using the molecular LDC instead of the LDC hydrochloride [11]. This is an interesting result since the hydrochloride LDC is more hydrophilic and therefore has low chemical affinity with the polymeric Strat-M membrane matrix. In this way, the PL407 + IPM ORGs improved the LDC permeation flux. It is also worth mentioning that the flux values for the ORGs evaluated in this work are about 10-fold higher when compared to formulations containing only IPM and LDC, when tested in human skin [55]. In fact, those differences observed on LDC permeation kinetics can be attributed to membrane type and specially the oil formulations composition. In another study, LDC permeation kinetics parameters were investigated across lanolin-based artificial membranes, reporting similar permeability coefficient values to those obtained here [56]. However, low permeability coefficient values were obtained for LDC from ORGs composed of PL40730% associated with oleic acid and lanolin, indicating that the incorporation of waxes and free fatty acids into the oil phase can reduce the drug permeation [11]. In a similar report, only 50% of LDC concentration applied was permeated across lanolin-added Strat-M artificial membrane [57]. 

Hence, the ORGs were characterized as highly structured gels, as concluded from the rheology results. Slow and controlled LDC permeation profiles from the PL407 + IPM was then expected from the LDC permeation results. Furthermore, it was not possible to identify m-CUR in the receptor medium after the 48 h of permeation analysis. This result demonstrates the high affinity of the m-CUR with the Strat-M membrane, corroborating the hydrophobic interactions observed between the membrane and the PL407 + IPM matrix [58]. 

**Figure 6:**
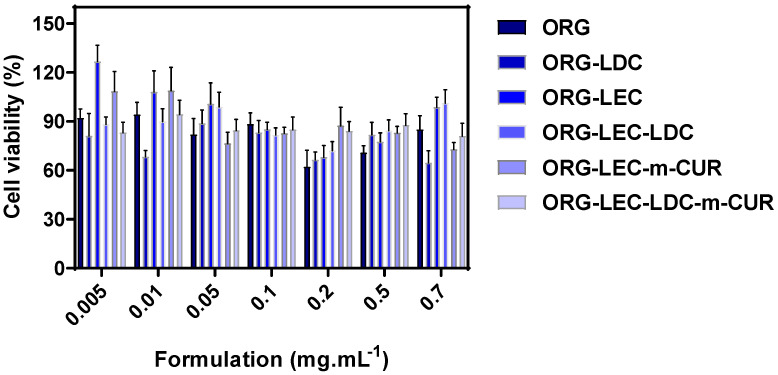
Cell viability percentage determined after HaCat cells treatment with ORG formulations (*n* = 6/concentration) by using the Methylthiazolyldiphenyl-tetrazolium bromide (MTT) reduction test.

In vitro cell viability assays were carried out in HaCat keratinocytes for assessing the possible cytotoxic effects evoked by ORGs and their different compositions, when evaluated by the MTT reduction test. Results are displayed in Figure 6. In general, most of the formulations are shown to be potentially non-toxic since the percentage of viable cells was from 101.3 to 81.2% for ORGS containing LEC and m-CUR, respectively. The lowest cell viability was observed for ORG-LDC, which can be explained by the possible high drug amounts available into the hydrophilic matrix. On the contrary, the presence of LEC increased the cell viability, probably due to the gradual drug release evoked by the association of both components into the oil phase (IPM and LEC), resulting in possible drug retention. Similar findings have been also reported for ORGs formulations with oil phases composed of lanolin/oleic acid and oleic acid in association with binary PL-based aqueous phase [11,58].

### 3.7. Epidermis Structural Analysis

**Figure 7:**
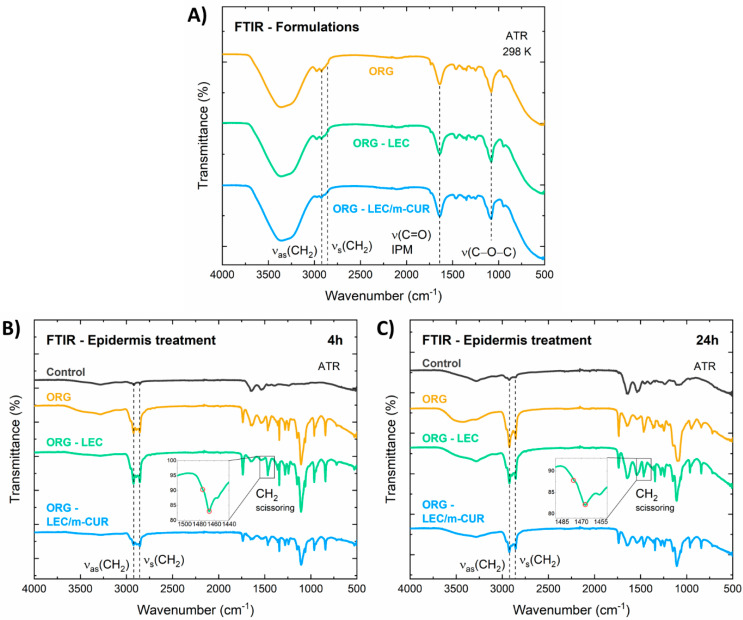
ATR-FTIR characterization of the (**A**) ORG, ORG-LEC, and ORG-LEC/m-CUR formulations, as well as the stratum corneum (SC) samples treated with these formulations for (**B**) 4 h and (**C**) 24 h. FTIR of a SC control sample (without ORG treatment) was also displayed.

Before evaluating the influence of ORGs into the epidermis structure, all formulations were characterized by FTIR. Additionally, the chemical interactions between the ORGs without LDC and porcine ear epidermis were also studied by employing the attenuated total reflectance (ATR) method (Figure 7).

In the FTIR spectra of the isolated ORGs (Figure 7A), one can appoint three main absorption bands assigned to the PL407 structure: (i) symmetrical (2858 cm^−1^), (ii) asymmetrical (2922 cm^−1^) stretching of CH_2_ groups, and (iii) C–O–C stretching at 1080 cm^−1^. In addition, a C=O carbonyl stretching absorption band can also be spotted at 1641 cm^−1^ [59], which arises from the IPM oil phase. The broad absorption band centered at 3350 cm^−1^ corresponds to the O–H vibrations from water molecules.

The effects induced in the epidermis by the treatment with the different ORG formulations were investigated, after 4 and 24 h of treatment (Figure 7B,C). According to Boncheva et al., the CH_2_ scissor bands at around ~1460 cm^−1^, together with the symmetric CH_2_ stretching at ~2855 cm^−1^, consist in powerful probes to investigate structural changes in the stratum corneum [28]. More specifically, the frequency and bandwidth of such bands are indicators for the conformational order of the SC lipid chain; the increase in the rotational movement of the alkyl chains during the transition from orthorhombic (OR) to hexagonal (HEX) structure, as well as the increase in the isomeric gauche effect on the alkyl chains during the transition from HEX to liquid crystal (LIQ), causes the CH_2_ absorption bands to broaden and to shift towards higher wavenumbers. In summary, disorganizing/fluidifying the SC structure leads to band broadening and shifting to higher energies.

In the FTIR spectrum of the control sample, the IR absorption band related to the symmetric CH_2_ stretching was identified at 2850 cm^−1^ (Figure 7B), which indicates the predominance of the HEX phase in the SC, mixed with an OR phase [28], characteristic of pig ear skin. For the epidermis treated with the ORGs, the symmetric CH_2_ stretching bands were identified around 2855 cm^−1^, indicating a fluidification process, i.e., the LIQ structure being the major phase in the SC. For all samples, after 24 h of application, the symmetric CH_2_ stretching bands were found in 2854–2855 cm^−1^ (Figure 7C), retaining the predominance of the LIQ structure. The wavenumber position and bandwidth for the CH_2_ scissor bands corroborated these results, once the two peaks at 1467 and 1475 cm^−1^ were shown to merge as a result of the ORG treatment (insets in Figure 7B,C), demonstrating a transition from HEX to LIQ structural organization.

**Figure 8:**
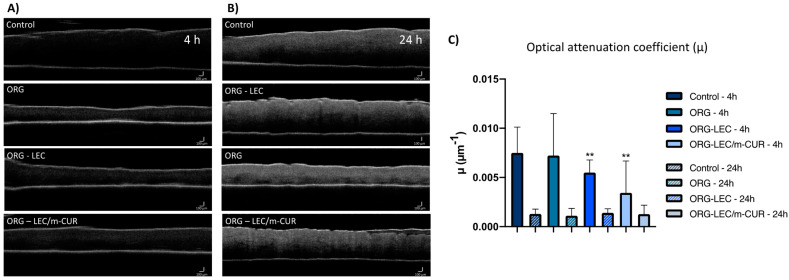
Optical Coherence Tomography (OCT) scans of dermatomed pig ear skin treated with ORGs for (**A**) 4 h and (**B**) 24 h, and (**C**) optical attenuation coefficient. Data are presented as mean ± SD (*n* = 3). Statistical differences by one-away ANOVA test relative to control vs. ORG-LEC and control vs. ORG-LEC/m-CUR, where *p* < 0.01 (**).

The impacts of ORGs treatment on the epidermis were also investigated by optical coherence tomography (OCT), an innovative technique for the morphological characterization of skin samples since it is non-invasive and provides images with micrometric scale depth and spatial resolution [60,61]. Optical attenuation coefficient (OAC), also called extinction coefficient or total attenuation, is an important quantitative parameter that can be extracted from OCT images [62,63]. As the OCT light attenuates along its path due to absorption and scattering processes, quantifying such attenuation considerably enhances image contrast in areas that otherwise would be difficult to distinguish. This parameter can be estimated from OCT data, and it is particularly important in the study of hard tissues, such as bones, as well as in skin imaging, facilitating structural segmentation and visualization [63]. Aiming at skin characterization, it is worth mentioning that filamentous proteins are the main source of light scattering in the skin, therefore the dermis (rich in collagen) and the epidermis (rich in keratin) show different values of OACs [60]. In this context, the OAC for each A-scan (µ) was calculated from the OCT images (Figure 8A,B) and the results were expressed in the graphs of Figure 8C. 

Results from OCT data treatment show that the optical attenuation coefficient (OAC) decreases in 4 h for the skin treated with ORGs containing the additives LEC and m-CUR, with *p* < 0.05 statistical difference relative to the control (Figure 8A). A lower OAC can be understood as a sparser distribution of collagen structures, consequently indicating a higher water content. In addition, collagen fibers could be dispersed among other water-absorber high-molecular-weight molecules present in the extracellular matrix, such as hyaluronic acid [64]. When light is scattered through structures such as keratin and dense collagen, e.g., scar tissue, they appear bright on OCT images. It is therefore noticeable from the decreasing OAC values (Figure 8C) that ORGs promote hydration of the analyzed skin samples. This phenomenon may be associated with modifications in the keratin and collagen fiber structures due to two main causes: (i) the water-occlusive effect caused by the three-dimensional structure of poloxamers—occlusion helps preserve natural moisturizing factors to confine water molecules locally; and (ii) the presence of LEC and m-CUR additives intensified the observed phenomenon—in the case of LEC, due to the affinity of the phospholipid for water molecules, and in the case of m-CUR, by a probable intermolecular interaction with hydrophobic components which enhanced the occlusion.

After 24 h, however, no statistical difference in the OAC parameter was observed among the samples (Figure 8B). All calculated OCAs were considerably low, including that of the control sample, which may indicate a degradation of skin structures. Ex vivo samples tend to be more sensitive to external factors such as temperature and relative humidity due to its thickness and lack of functioning cutaneous circulation when compared to in vivo samples [65]. In this way, we concluded that 24 h is too long considering the integrity of skin samples for OCT analyses.

## 4. Conclusions

The physical chemical properties of PL407-based organogels (ORGs), as well as their interaction with the skin, were thoroughly explored, aiming at dual drug delivery systems for topical applications. This is a comprehensive work which comprises the synthesis of new bioactive molecules, together with the preparation and characterization of ORG formulations, and the ex vivo response of skin models in terms of structural organization and drug permeation profiles. To the best of our knowledge, this is the first report of a monoketone curcuminoid (m-CUR) synthesized by a Claisen–Schmidt condensation reaction via NbCl_5_ acid catalysis.

Our results point to a high versatility for the ORG systems since its structural properties can be modulated by changing its composition, i.e., by the incorporation of different additives, obtaining formulations for both transdermal (high permeation flux) and topical applications (in which drugs remain trapped in the outer layers of the skin). For instance, soy lecithin (LEC) additive was demonstrated to increase the organizational degree of the ORGs three-dimensional lattice, leading to a slower drug permeation flux. Such ORGs are therefore interesting for topical applications such as scar healing and treatment of painful injuries, considering the drug association incorporated into them. It is worth noting that the presence of additives did not cause substantial changes the ORG supramolecular structure as showed by SANS results, thus preserving the long-term stability for all formulations.

Infrared spectroscopy (FTIR) study of porcine ear epidermis treated with the ORGs revealed a displacement of the symmetrical and asymmetrical stretching bands of C–H bonds to higher wavenumbers when compared to the control samples. This result indicates that stratum corneum lipids undergo a hexagonal-to-liquid crystal phase transition after treatment with ORGs, which agrees with previous works on skin structure. Moreover, from optical coherence tomography (OCT), we observed that ORGs containing additives (LEC and m-CUR) tend to decrease the skin’s optical attenuation coefficient within 4 h of ORG treatment. This points to structural modifications in the skin, mainly in the organization of filamentous proteins, such as keratin and collagen. FTIR and OCT were then proved to be complementary tools for ex vivo skin structural evaluation, more specifically regarding the study of lipid organization in the stratum corneum and fibrous proteins in the superficial skin layers, which are in contact with the formulations. Finally, the presence of m-CUR in ORGs promoted structural changes in both organogels and skin components. Additional studies on vibrational spectroscopic could unravel the mechanism behind such structural changes to further explain synergistic pharmacological effects between m-CUR and LDC, in association with future in vivo studies.

## Figures and Tables

**Table 1:** Prepared organogels (ORG) formulations. All ORG are composed of an aqueous phase of PL407 30% *w*/*v* and an oil phase of isopropyl myristate (IPM). LDC: Lidocaine hydrochloride, LEC: soy lecithin, m-CUR: synthesized monoketone curcuminoid.

Formulations	Additives	Composition (%, *w*/*v*)
ORG	-	PL407 (30%) and IPM (1%)
ORG-LDC	LDC	ORG composition + LDC (2%)
ORG-LEC	LEC	PL407 (30%) and IPM (1%) + LEC (2%)
ORG-LDC/LEC	LDC + LEC	ORG-LEC composition + LDC (2%)
ORG-LEC/m-CUR	LEC + m-CUR	ORG-LEC composition + m-CUR (0.02%)
ORG-LDC/LEC/m-CUR	LDC + LEC + m-CUR	ORG-LEC composition + LDC (2%) and m-CUR (0.02%)

**Table 2:** Phase transition temperatures (T_peak_), together with the initial (T_onset_) and final (T_endset_) transition temperatures, and the micellization enthalpy variation (ΔH_m_) for all ORGs.

Formulation	T_onset_ (°C)	T_peak_ (°C)	T_endset_ (°C)	ΔH_m_ (J·g^−1^)
ORG	3.9	9.4	12.4	8.3
ORG-LDC	3.9	9.2	12.8	7.6
ORG-LEC	3.9	10.0	13.3	8.0
ORG-LDC/LEC	3.8	9.4	12.4	8.8
ORG-LEC/m-CUR	3.8	9.3	12.6	7.4
ORG-LDC/LEC/m-CUR	3.8	8.9	11.9	7.0

**Table 3:** Rheological parameters: sol–gel transition temperature (T_sol-gel_, G′ (elastic) and G″ (viscous) moduli, and viscosity (η*) at 32.5 °C for the PL407 + IPM ORG formulations.

Formulation	T_sol-gel_ (°C)	G′ (Pa)	G″ (Pa)	G′/G″	η* (mPas.s) × 10^3^
ORG	21.13 ± 0.80	5066	257.8	19.6	807.4
ORG-LDC	23.07 ± 1.29	4457	347.2	12.8	711.5
ORG-LEC	25.82 ± 0.10	4367	188.3	23.2	695.6
ORG-LDC/LEC	23.37 ± 0.41	5892	273.6	21.5	938.7
ORG-LEC/m-CUR	30.46 ± 0.14	3474	174.0	20.0	553.6
ORG-LDC/LEC/m-CUR	29.14 ± 0.76	6453	270.1	23.9	1028

Note: T_sol-gel_ (°C) data expressed as mean ± standard deviation, *n* = 3/experiment.

**Table 4:** 1/q values (Å) at 40 °C, as well as the assigned crystal space groups to the different cubic phases found in the ORG samples from SANS analyses.

Formulation	1/q (Å) at 40 °C	Crystal Space Group
ORG	23.6 (0.8)	$Pm\bar{3}n\;$(#223)
	28.9	$Fd\bar{3}m\;$(#227)
ORG-LDC	23.6 (0.7)	$Pm\bar{3}n$
	28.9	$Fd\bar{3}m$
ORG-LEC	24.3 (0.8)	$Pm\bar{3}n$
	29.8	$Fd\bar{3}m$
ORG-LDC/LEC	23.6 (0.7)	$Pm\bar{3}n$
	28.9	$Fd\bar{3}m$
	40.8	$Ia\bar{3}d\;$(#230)
ORG-LEC/m-CUR	23.6 (0.7)	$Pm\bar{3}n$
	28.9	$Fd\bar{3}m$
	40.8	$Ia\bar{3}d$
ORG-LDC/LEC/m-CUR	23.6 (0.7)	$Pm\bar{3}n$
	28.9	$Fd\bar{3}m$
	40.8	$Ia\bar{3}d$

**Table 5:** Lidocaine hydrochloride (LDC) permeation parameters across the Strat-M^®^ membrane.

ORGs	Drug	Flux (μg·cm^−2^·h^−1^)	T_lag_ (h)	Permeability Coefficient (cm·h^−1^, ×10^−2^)
ORG-LDC	LDC	15.54 ± 1.27	8.82 ± 0.27	1.29 ± 0.11
ORG-LDC/LEC	LDC	19.92 ± 2.88	9.34 ± 0.03	1.66 ± 0.66
ORG-LDC/LEC/m-CUR	LDC	20.76 ± 1.66	9.28 ± 0.75	1.73 ± 0.20

## Data Availability

Data available on motivated request.

## Supplementary Materials

The following supporting information can be downloaded at: https://www.mdpi.com/article/10.3390/pharmaceutics14020293/s1. Figure S1: ^1^H-NMR spectrum of m-CUR; Figure S2: ^13^C-NMR spectrum of m-CUR; Figures S3 and S4: SANS spectra of ORG formulations recorded at 25 and 40 °C, respectively.
