# Supplementary material for: Monoketonic Curcuminoid-Lidocaine Co-Deliver Using Thermosensitive Organogels: From Drug Synthesis to Epidermis Structural Studies
Source: Pharmaceutics. 2022 Jan 27;14(2):293. doi: 10.3390/pharmaceutics14020293 (PMC8879257; doi:10.3390/pharmaceutics14020293)
Supplement: Supplementary file 1 [file pharmaceutics-14-00293-s001.zip › pharmaceutics-1547210-supplementary.pdf]

pharmaceutics

Supplementary Materials: Monoketonic curcuminoid-lidocaine co-deliver using thermosensitive organogels: from drug synthesis to epidermis structural studies

Aryane A. Vigato, Ian P. Machado, Matheus del Valle, Patricia A. da Ana, Anderson F. Sepulveda, Fabiano Yokaichiya, Margareth K. K. D. Franco, Messias C. Loiola, Giovana R. Tófoli, Cintia Maria S. Cereda, Mirela I. de Sairre and Daniele R. de Araujo

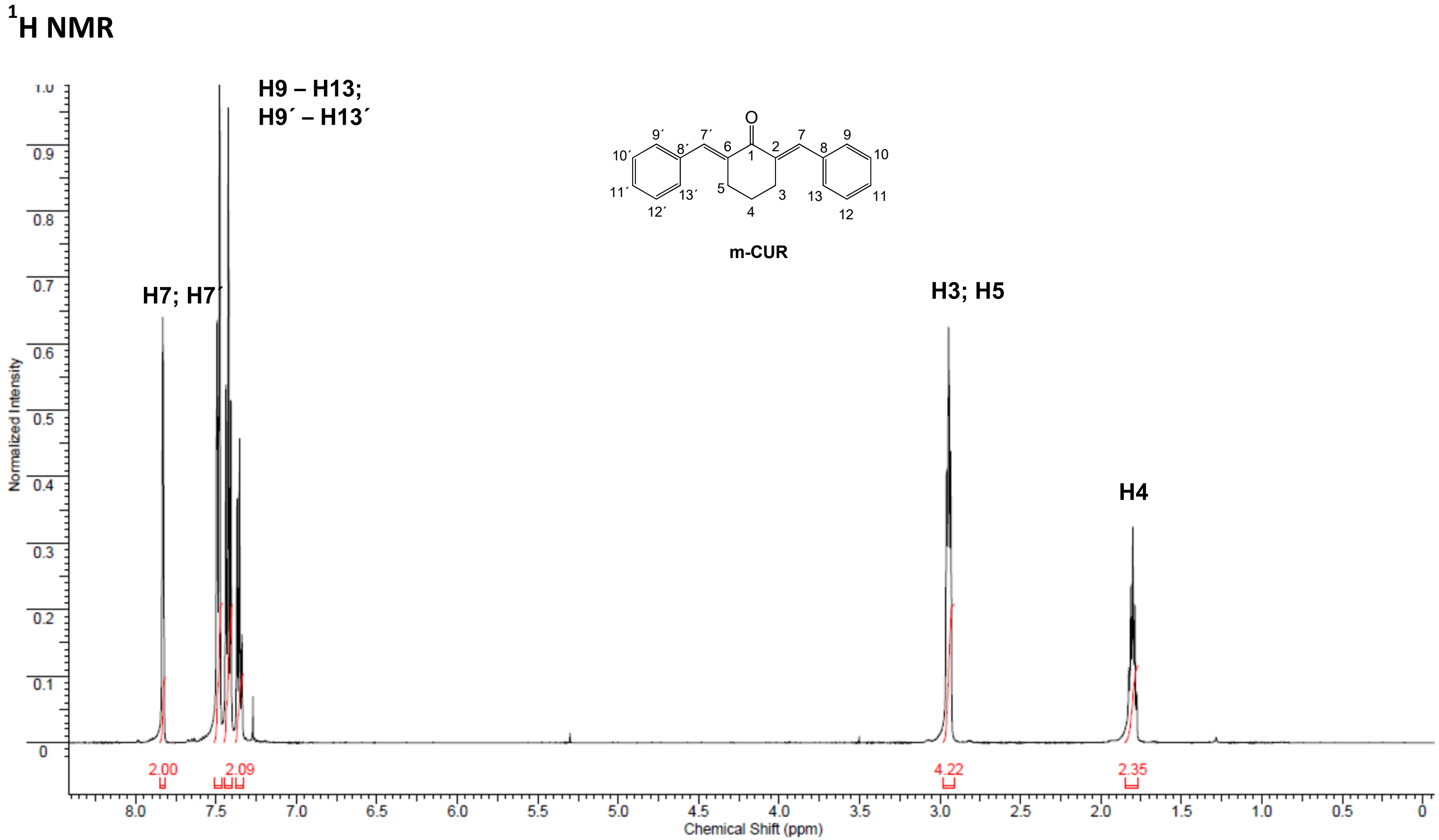

Figure S1. ^{1}H-NMR spectrum of m-CUR molecule in $CDCl_3$.

^{13}C NMR

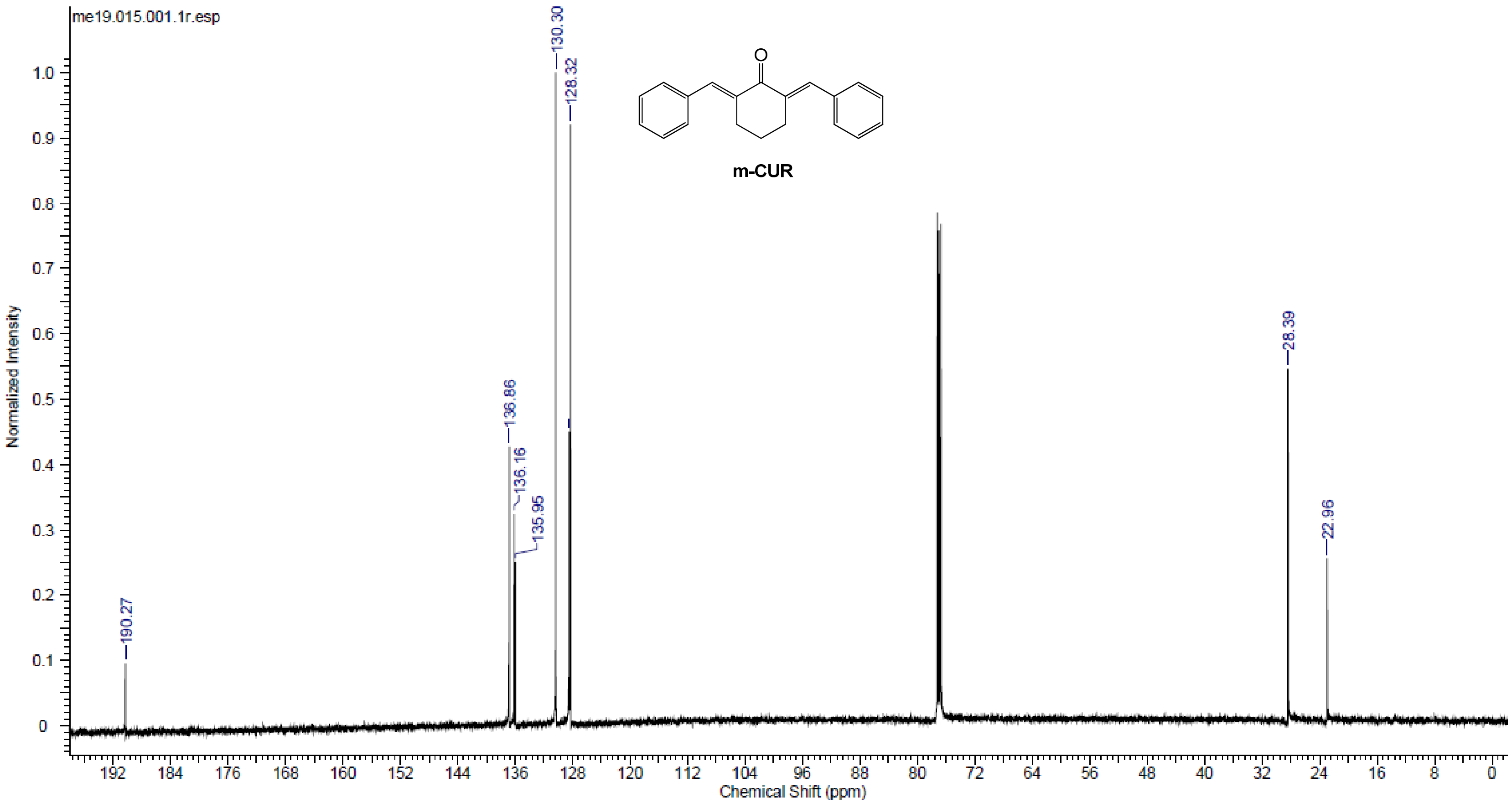

Figure S2. ^{13}C-NMR spectrum of m-CUR molecule in $CDCl_3$

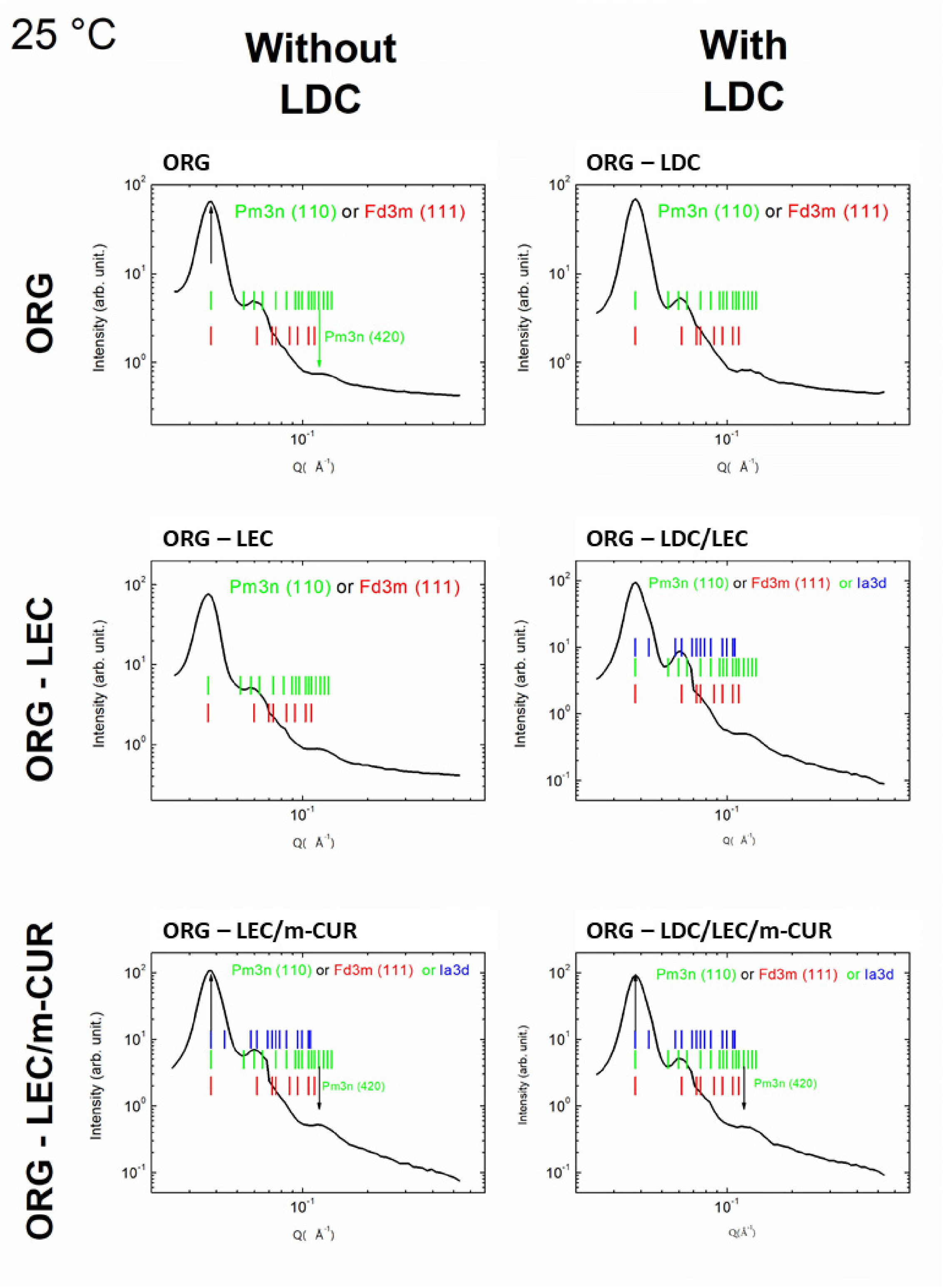

Figure S3. Small-angle neutron scattering (SANS) patterns of ORG formulations without (left) and with (right) lidocaine hydrochloride (LDC). 1/q parameter was fitted to found three different cubic structures – $Pm\bar{3}n$, $Fd\bar{3}m$, and $Ia\bar{3}d$. All measurements were performed at room temperature – 25 °C.

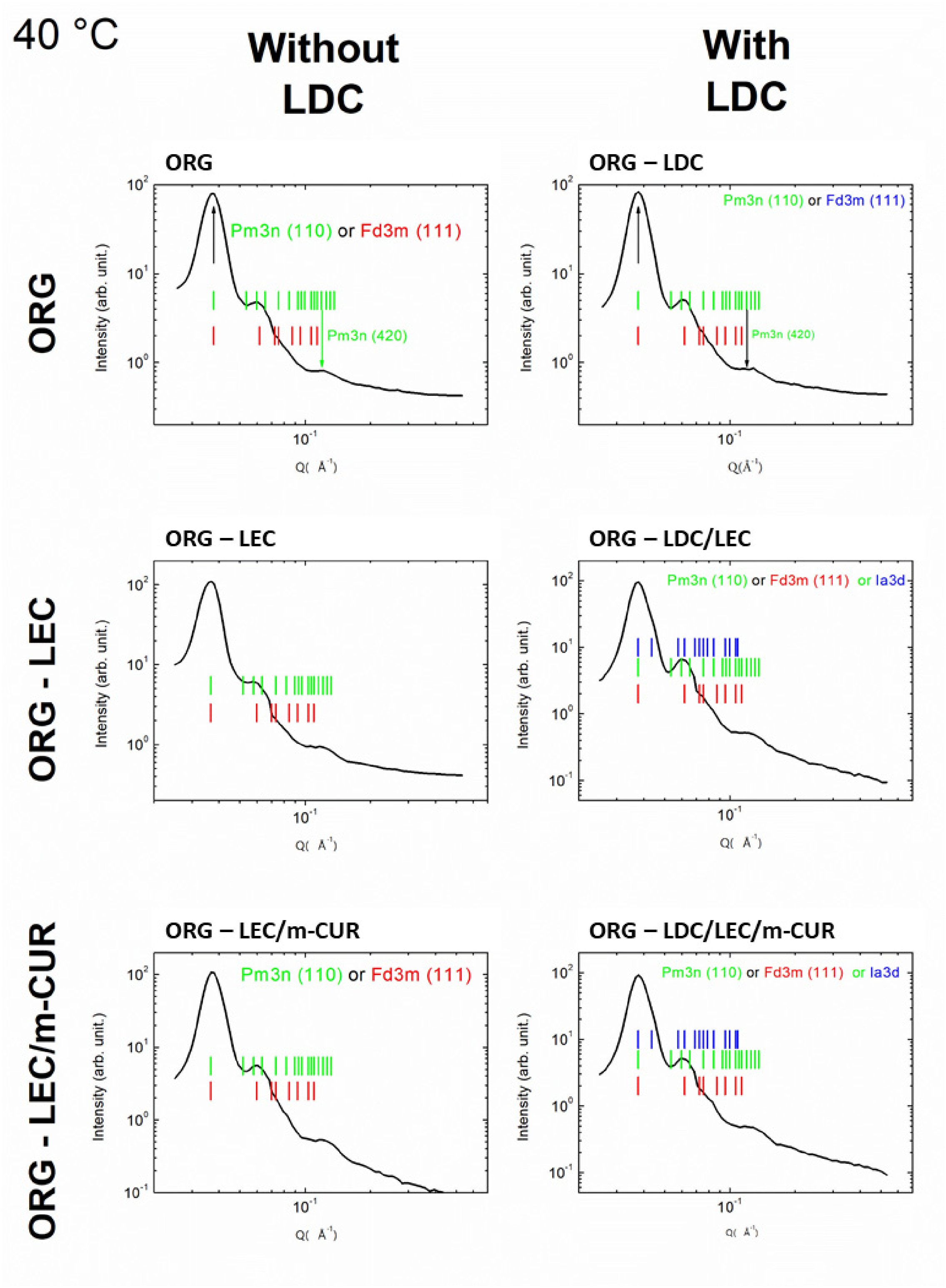

Figure S4. Small-angle neutron scattering (SANS) patterns of ORG formulations without (left) and with (right) lidocaine hydrochloride (LDC). In this case, measurements were performed at 40 °C.
